# Sensor Fault and Delay Tolerant Control for Networked Control Systems Subject to External Disturbances

**DOI:** 10.3390/s17040700

**Published:** 2017-03-28

**Authors:** Shi-Yuan Han, Yue-Hui Chen, Gong-You Tang

**Affiliations:** 1 Shandong Provincial Key Laboratory of Network Based Intelligent Computing, University of Jinan, Jinan 250022, China; yhchen@ujn.edu.cn; 2 College of Information Science and Engineering, Ocean University of China, Qingdao 266100, China; gtang@ouc.edu.cn

**Keywords:** fault tolerance control, disturbance rejection, sensor fault, delayed measurement, external disturbances

## Abstract

In this paper, the problem of sensor fault and delay tolerant control problem for a class of networked control systems under external disturbances is investigated. More precisely, the dynamic characteristics of the external disturbance and sensor fault are described as the output of exogenous systems first. The original sensor fault and delay tolerant control problem is reformulated as an equivalence problem with designed available system output and reformed performance index. The feedforward and feedback sensor fault tolerant controller (FFSFTC) can be obtained by utilizing the solutions of Riccati matrix equation and Stein matrix equation. Based on the designed fault diagnoser, the proposed FFSFTC is further reconstructed to compensate for the sensor fault and delayed measurement effects. Finally, numerical examples are provided to illustrate the effectiveness of our proposed FFSFTC with different cases with various types of sensor faults, measurement delays and external disturbances.

## 1. Introduction

Fault tolerant control has been viewed as an excellent control approach in modern industrial processes, which can provide stricter security and reliability performance for practical systems [1,2]. Meanwhile, networked control systems (NCSs) have been integrated into automatic control systems with the technologies of dependent actuators, sensors and microprocessors [3,4,5,6,7]. What corresponds with this is the frequency of sensor failures arising from real-time continuous monitoring data with high sampling rate [8,9]. The increased complexity of the systems and the availability of sensors introduce the necessity of fault detection technologies and fault tolerant control systems. Fortunately, effective sensor fault tolerant control strategies have been proposed to enhance the safety and reliability for industrial automatic systems in theory and application, including passive design [10,11] and active fault tolerant control [12,13,14] with low costs and flexible structures.

Since any sensor is subject to measurement delay, it is impossible to implement the accurate system state to design the fault tolerant control scheme. A great deal of attention has been focused on the compensation of delayed measurements, in which the observer techniques and the fault accommodation strategies play an important role in obtaining the goals of sensor fault tolerant control for NCSs. With regard to observer techniques, a local observer was proposed to provide the information of both state, uncertainty and fault information in [15]; a new observer-based reduced-order fault diagnoser construction approach and a dynamic self-restore fault-tolerant control law were designed based on an augmented system in [16]; and a nonlinear adaptive observer was designed to estimate the faulty parameters and designed a fault diagnosis scheme in [17] . For the fault accommodation strategies, a modified fault isolation filter for NCSs with multiple faults was designed to improve the resource utilization with graceful fault estimation performance degradation in [18]; the boundedness of all closed-loop signals were ensured by adopting the designed adaptive fault compensation control scheme for parametric strict feedback nonlinear systems in [19]; and the detection threshold was detected to detect the nonlinear fault, and designed a fault accommodation scheme using the function approximation technique in [20]. The fault tolerant control problem for NCSs with sensor fault and delay will be discussed from the perspective of the optimal control in this paper.

Focused on control performance and stability of industrial systems, the influence of inevitable external disturbance is also worthy of consideration [21,22,23,24]. In particular, it is necessary to integrate the disturbance rejection techniques into fault tolerant control scheme. However, only a few results were presented for the fault tolerant control problem under external disturbances. For instance, [25] introduced two robust observers to eliminate the effect of fault and disturbance and designed sliding mode control (SMC) scheme against faults and disturbances; an anti-disturbance fault tolerant control scheme was presented for a class of nonlinear delay systems with both faults and multiple disturbances in [26]; an online adaptive mechanism was constructed to estimate the unknown fault parameters, and designed an adaptive fault tolerant control scheme involved with a backstepping technique in [27]; both disturbance observer and fault estimation observer were proposed to design the novel fuzzy dynamic output feedback fault tolerant controller by using the estimation information in [28]. How to offset the external disturbances and compensate the sensor fault and delay simultaneously is one of the motivations of this article.

Based on the above statements, this paper deals with a sensor fault and delay tolerant control problem for NCSs subject to external disturbances. Under assumptions that the dynamic characteristics of external disturbance and sensor fault are known, the delayed output is reformed as an available output without explicit expression of delayed measurement. The performance index is transformed as a corresponding form so that the two-point-boundary-value (TPBV) problem with delayed and advanced items can be avoided. Thus, the feedforward and feedback sensor fault tolerant controller (FFSFTC) is derived from the solutions of the Riccati matrix equation and Stein matrix equation, in which a fault diagnoser is designed to make the proposed control scheme physical realizability. Taking the different kinds of sensor faults and external disturbances into consideration, illustrative examples are provided to prove the effectiveness of the FFSFTC under different measurement delays.

This paper is organized as follows. Section 2 formulates the sensor fault and delay tolerant control problem for NCSs. In Section 3, the main results are presented, which include the proposed FFSFTC and a fault diagnoser. Numerical examples are given to verify the effectiveness of the proposed control scheme in Section 4. Section 5 gives the conclusions.

## 2. Problem Formulation

Consider the following discrete generalized NCSs with delayed and faulty sensors under external disturbance:

(1) $$\left\{\begin{array}{c} x\left(k+1\right)=Ax\left(k\right)+Bu\left(k\right)+D_{v}v\left(k\right),\;k=0,\;1,\;2\;\ldots ,\\ y\left(k\right)=Cx\left(k-h_{s}\right)+D_{s}f_{s}\left(k-h_{s}\right),\;k=h_{s},\;h_{s}+1,\;h_{s}+2\;\ldots ,\\ x\left(0\right)=x_{0},\\ y\left(k\right)=0,\;k=0,\;1,\;\ldots ,\;h_{s}-1, \end{array}\right.$$

where $x\left(k\right)\in R^{n},u\left(k\right)\in R^{m}$ and $v\left(k\right)\in R^{p}$ denote the state vector, the control input and the external disturbance for NCSs, respectively. $f_{s}\left(k\right)\in R^{q}$ represents the fault signal vector, $h_{s}$ denotes a constant measurement delay of the sensor, and $y\left(k\right)\in R^{l}$ represents the system measured output; all $A,B,C,D_{v}$ and $D_{s}$ are known constant matrices with appropriate dimensions.

The dynamic characteristics of the external disturbance signal v(k) can be described by the following exogenous system:

(2) $$\left\{\begin{array}{c} w\left(k+1\right)=G_{v}w\left(k\right),\\ v\left(k\right)=H_{v}w\left(k\right), \end{array}\right.$$

where $w\left(k\right)\in R^{r_{v}}$ denotes the state vector for external disturbances. $G_{v}$ and $H_{v}$ are known constant matrices with appropriate dimensions. The exogenous system (2) can describe various types of external disturbances, such as step signal, period signal, attenuation signal and other extensive persistent disturbances in discrete form.

The dynamic characteristics of the sensors fault signal $f_{s}\left(k\right)$ can be represented as:

(3) $$\left\{\begin{array}{c} \varphi \left(k+1\right)=G_{s}\varphi \left(k\right),\;k=k_{0},\;k_{0}+1,\;k_{0}+2,\;\ldots ,\\ f_{s}\left(k\right)=F_{s}\varphi \left(k\right),\;k=0,\;1,\;2,\;\ldots ,\\ \varphi \left(k\right)=0,\;k=0,\;1,\;2,\;k_{0}-1,\\ \varphi \left(k_{0}\right)=\varphi_{0}, \end{array}\right.$$

where $\varphi \in R^{r_{f}}$ is the state vector of the sensor fault, the occurrence time $k_{0}$ and initial state $\varphi_{0}$ are with unknown values. Meanwhile, the exogenous (3) can describe the diverse sensor faults with known dynamic characteristics and unknown magnitudes and phases. In order to diagnose the sensor fault and design a sensor fault and delay tolerant controller for NCSs, the following assumptions are given.

**Assumption** **1.** The pair (A,B) is completely controllable and the pair (C,A) is completely observable.

**Assumption** **2.** The pairs $\left(H_{v},G_{v}\right)$ in (2) and $\left(F_{s},G_{s}\right)$ in (3) are completely observable.

**Assumption** **3.** For any eigenvalue $\lambda_{i}$ of $G_{v}$ and $\lambda_{j}$ of $G_{s}$ satisfying $\left|\lambda_{i}\right|\leq 1$ and $\left|\lambda_{j}\right|\leq 1$, the persistent disturbances v(k) and sensor fault $f_{s}\left(k\right)$ are stable but may not be asymptotically stable.

The aim of this paper is formulated to design a dynamic fault diagnoser to diagnose sensor faults thereby designing an optimal sensor fault and delay tolerant control scheme $u^{*}\left(k\right)$ for system (1) subject to external disturbance (2) and sensor fault (3) to minimum the following average quadratic performance index:

(4) $$J=\lim_{N\to \infty }\frac{1}{2N}\sum_{k=0}^{N}\left[y^{T}\left(k\right)Qy\left(k\right)+u^{T}\left(k\right)Ru\left(k\right)\right],$$

where $Q=C^{T}C\in R^{l\times l}$ is a positive semi-definite matrix and R>0 is a constant.

For the classic optimal control problem, the following Hamilton function will be obtained:

(5) $$\begin{array}{c} H\left(•\right)=\frac{1}{2}\left[x^{T}\left(k-h_{s}\right)C^{T}QCx\left(k-h_{s}\right)+x^{T}\left(k-h_{s}\right)C^{T}QD_{s}f_{s}\left(k-h_{s}\right)+u^{T}\left(k\right)Ru\left(k\right)\right.\\ \left.+f_{s}^{T}\left(k-h_{s}\right)D_{s}^{T}QD_{s}f_{s}\left(k-h_{s}\right)\right]+\lambda^{T}\left(k+1\right)\left[Ax\left(k\right)+Bu\left(k\right)+D_{v}v\left(k\right)-x\left(k+1\right)\right]. \end{array}$$

It is obvious that this function includes some delay items. When transferring the two-point-boundary- value (TPBV) problem, the advanced and delayed items are unavoidable. This is a motivation of this paper to avoid the TPBV problem with advanced and delayed items.

To obtain the main results, the following lemma is given first.

**Lemma** **1.** *Let $A_{1}\in R^{n\times n},B_{1}\in R^{m\times m}$, and $X\in R^{n\times m}$. The matrix equation*

(6) $$A_{1}XB_{1}-X=C_{1}$$

*has a unique solution X if and only if*

(7) $$\lambda_{i}\left(A_{1}\right)\times \lambda_{j}\left(B_{1}\right)\neq 1,\;i=1,\ldots ,n,\;j=1,\ldots ,m,$$

*where λ(·) denote eigenvalues of matrix [29].*

## 3. Design of Optimal Sensor Fault and Delay Tolerant Control Scheme

In this section, the original sensor fault and delay tolerant control problem is reformulated as an equivalent form first. Then, an optimal feedforward and feedback sensor fault and delay tolerant control scheme will be designed.

### 3.1. Reformulation of the Sensor Fault and Delay Tolerant Control Problem

Accurate system state views are one essential factor in designing the sensor fault and delay tolerant control scheme. However, system output y(k) presented system (1) is dependent on the measurement delay and sensor fault. From the optimal control point of view, seeking the solution for the TPBV problem with advanced and delayed items is very difficult. In what follows, an available output and a reformed performance index will be designed to transform the original TPBV problem.

Combining systems (1) and (2) with an augmented vector $z\left(k\right)={\left[\begin{array}{cc} x^{T}\left(k\right) & w^{T}\left(k\right) \end{array}\right]}^{T}$, the following augmented system can be generated as

(8) $$\left\{\begin{array}{c} z\left(k+1\right)=A_{1}z\left(k\right)+B_{1}u\left(k\right),\\ y\left(k\right)=\left\{\begin{array}{c} 0,\;k=0,\;1,\;2,\;\ldots ,h_{s}-1,\\ \bar{C}z\left(k-h_{s}\right)+D_{s}f_{s}\left(k-h_{s}\right),\;k=h_{s},\;h_{s}+1,\;\ldots , \end{array}\right. \end{array}\right.$$

where

(9) $$A_{1}=\left[\begin{array}{cc} A & H_{v}G_{v}\\ 0 & G_{v} \end{array}\right],B_{1}=\left[\begin{array}{c} B\\ 0 \end{array}\right],\bar{C}=\left[\begin{array}{cc} C & 0 \end{array}\right].$$

The solution of system (8) can be described as

(10) $$\left\{\begin{array}{c} z\left(k\right)=A_{1}^{k}z\left(0\right)+\sum_{i=0}^{k-1}A^{k-i-1}B_{1}u\left(i\right),\;k=0,\;1,\;2,\;\ldots ,\\ y\left(k\right)=\left\{\begin{array}{c} 0,\;k=0,\;1,\;2,\;\ldots ,h_{s}-1,\\ C_{1}\left[A_{1}^{k}z\left(0\right)+\sum_{i=0}^{k-h_{s}-1}A_{1}^{k-i-1}B_{1}u\left(i\right)\right]+D_{1}\varphi \left(k\right),\;k=h_{s},\;h_{s}+1,\;\ldots , \end{array}\right. \end{array}\right.$$

where $C_{1}=\bar{C}A_{1}^{-h_{s}}$ and $D_{1}=D_{s}F_{s}G_{s}^{-h_{s}}$. Thus, the available output $y_{1}\left(k\right)$ is defined as follows:

(11) $$y_{1}\left(k\right)=y\left(k\right)+C_{1}\gamma \left(k,u\right),$$

where $\gamma \left(k,u\right)=\sum_{i=k-h_{s}}^{k-1}A_{1}^{k-i-1}B_{1}u\left(i\right)$. Thus, system (1) can be rewritten as

(12) $$\left\{\begin{array}{c} z\left(k+1\right)=A_{1}z\left(k\right)+B_{1}u\left(k\right),\\ y_{1}\left(k\right)=\left\{\begin{array}{c} C_{1}z\left(k\right),\;k=0,\;1,\;2,\;\ldots ,\;h_{s}\\ C_{1}z\left(k\right)+D_{1}\varphi \left(k\right),\;k=h_{s}+1,\;h_{s}+2,\;h_{s}+3,\;\ldots , \end{array}\right.\\ y\left(k\right)=\left\{\begin{array}{c} C_{1}z\left(k\right)-C_{1}\gamma \left(k,u\right),\;k=0,\;1,\;2,\;\ldots ,\;h_{s}\\ C_{1}z\left(k\right)+D_{1}\varphi \left(k\right)-C_{1}\gamma \left(k,u\right),\;k=h_{s}+1,\;h_{s}+2,\;h_{s}+3,\;\ldots . \end{array}\right. \end{array}\right.$$

Inevitably, the quadratic performance index (4) must be transformed into the corresponding form with respect to the augmented system (8). In what follows, we will construct a corresponding performance index for original performance index (4).

Replacing y(k) as $y_{1}\left(k\right)$ in (4), we have

(13) $$\begin{array}{c} J=\lim_{N\to \infty }\frac{1}{2N}\sum_{k=0}^{N}z^{T}\left(k\right)C_{1}^{T}QC_{1}z\left(k\right)+2z^{T}\left(k\right)C_{1}^{T}QD_{1}\varphi \left(k\right)+u^{T}\left(k\right)Ru\left(k\right)\\ \;\left.-2z^{T}\left(k\right)C_{1}^{T}QC_{1}\gamma \left(k,u\right)-2\varphi^{T}\left(k\right)D_{1}^{T}QC_{1}\gamma \left(k,u\right)+\gamma^{T}\left(k,u\right)C_{1}^{T}QC_{1}\gamma \left(k,u\right)\right\}. \end{array}$$

Developing and analyzing the above equation along with *k* under constraints (2) and (3), one gets

(14) $$\left\{\begin{array}{c} \sum_{k=0}^{\infty }z^{T}\left(k\right)C_{1}^{T}QC_{1}\gamma \left(k,u\right)=\sum_{k=0}^{\infty }\left\{z^{T}\left(k\right)Q_{zu}u\left(k\right)+u^{T}\left(k\right)Q_{uu}u\left(k\right)\right.\\ \left.\;+2\left(\sum_{i=1}^{h_{s}-1}{\left(\sum_{j=i}^{h_{s}-1}A_{1}^{j-i}B_{1}u\left(k+h_{s}-j\right)\right)}^{T}C_{1}^{T}QC_{1}A^{i}B_{1}\right)u\left(k\right)\right\}\\ \sum_{k=0}^{\infty }\varphi^{T}\left(k\right)D_{1}^{T}QC_{1}\gamma \left(k,u\right)=\sum_{i=h_{s}}^{\infty }\varphi^{T}\left(k\right)Q_{\varphi u}u\left(k\right),\\ \sum_{k=0}^{\infty }\gamma^{T}\left(k,u\right)C_{1}^{T}QC_{1}\gamma \left(k,u\right)=\sum_{k=0}^{\infty }\left\{u^{T}\left(k\right)Q_{uu}u\left(k\right)+\right.\\ \left.\;+2\left(\sum_{i=1}^{h_{s}-1}{\left(\sum_{j=i}^{h_{s}-1}A_{1}^{j-i}B_{1}u\left(k+h_{s}-j\right)\right)}^{T}C_{1}^{T}QC_{1}A^{i}B_{1}\right)u\left(k\right)\right\}, \end{array}\right.$$

where

(15) $$\begin{array}{c} Q_{\varphi u}=\sum_{i=1}^{h_{s}}{\left(D_{1}G_{s}^{i}\right)}^{T}QC_{1}A_{1}^{i-1}B_{1},\\ Q_{zu}=\sum_{i=1}^{h_{s}}{\left(C_{1}A_{1}^{i}\right)}^{T}QC_{1}A_{1}^{i-1}B_{1},Q_{uu}=\sum_{i=0}^{h_{s}-1}{\left(C_{1}A_{1}^{i}B_{1}\right)}^{T}QC_{1}A_{1}^{i}B_{1}. \end{array}$$

Thus, the original performance index (4) can be reformed as the corresponding form with respect to the argument system (11), which is described as

(16) $$\begin{array}{c} J=\lim_{N\to \infty }\frac{1}{2N}\sum_{k=0}^{N}\left\{z^{T}\left(k\right)C_{1}^{T}QC_{1}z\left(k\right)+2z^{T}\left(k\right)C_{1}^{T}QD_{1}\varphi \left(k\right)\right.\\ \;\left.-2z^{T}\left(k\right)Q_{zu}u\left(k\right)-2\varphi^{T}\left(k\right)Q_{\varphi u}u\left(k\right)+u^{T}\left(k\right)R_{1}u\left(k\right)\right\}, \end{array}$$

where $R_{1}=R-R_{uu}$.

The original sensor fault and delay tolerant control problem are reformulated to design an optimal sensor fault and delay tolerant control scheme $u^{*}\left(k\right)$ for system (12) to minimize the reformed performance index (16) under the constraint condition (3).

### 3.2. Design of Optimal Sensor Fault and Delay Tolerant Control Scheme Based on the Sensor Fault Diagnoser

The following Theorem provides a method to propose an optimal sensor fault and delay tolerant control scheme based on a designed sensor fault diagnoser.

**Theorem** **1.** *Considering the dynamic characteristics of sensor fault (3), a dynamic fault diagnoser is designed to diagnose the sensor fault, which can be described as*

(17) $$\left\{\begin{array}{c} \tilde{\varphi }\left(k+1\right)=\left(G_{s}-K_{e}F_{s}\right)\tilde{\varphi }\left(k\right)+K_{e}f_{s}\left(k\right),\\ {\tilde{f}}_{s}\left(k\right)=F_{s}\tilde{\varphi }\left(k\right), \end{array}\right.$$

*where $\tilde{\varphi }\left(k\right)$ denotes the fault diagnoser state, $K_{e}$ represents the fault diagnoser feedback gain matrix, and ${\tilde{f}}_{s}\left(k\right)$ is a prediction sensor fault signal.*

*Considering the sensor fault and delay tolerant control problem for a class of NCSs (1) under the constraints of the persistent disturbances (2) and the sensor fault (3) with respect to quadratic performance index functional (4), under Assumptions 1, 2 and 3, there exists a unique optimal sensor fault and delay tolerant control scheme in the form as*

(18) $$u^{*}\left(k\right)=-R_{1}^{-1}\left(B_{1}^{T}{\bar{A}}^{-T}\left(P_{1}-Q_{1}\right)-Q_{zu}^{T}\right)z\left(k\right)+\left(B_{1}^{T}{\bar{A}}^{-T}\left(P_{2}-Q_{2}\right)-Q_{\varphi u}^{T}\right)\tilde{\varphi }\left(k\right),$$

*where $P_{1}$ is the unique positive definite solution of the following Riccati matrix equation*

(19) $$P_{1}=Q_{1}+{\bar{A}}^{T}P_{1}\bar{A}-{\bar{A}}^{T}P_{1}B_{1}{\left(R_{1}+B_{1}^{T}P_{1}B_{1}\right)}^{-1}B_{1}P\bar{A}.$$

*$P_{2}$ is the unique solution of the following Stein matrix equation*

(20) $$\begin{array}{c} P_{2}=Q_{2}+{\bar{A}}^{T}P_{2}G_{s}+{\bar{A}}^{T}P_{1}{\left(I+B_{1}R_{1}^{-1}B_{1}^{T}P_{1}\right)}^{-1}B_{1}R_{1}^{-1}Q_{\varphi u}^{T}\\ \;+{\bar{A}}^{T}\left(I-P_{1}{\left(I+B_{1}R_{1}^{-1}B_{1}^{T}P_{1}\right)}^{-1}B_{1}R_{1}^{-1}B_{1}^{T}\right)P_{2}G_{s}, \end{array}$$

*with $\bar{A}=A_{1}+B_{1}R_{1}^{-1}Q_{zu}^{T},Q_{1}=C_{1}^{T}QC_{1}-Q_{zu}R_{1}^{-1}Q_{zu}^{T},Q_{2}=C_{1}^{T}QD_{1}-Q_{zu}R_{1}^{-1}Q_{\varphi u}^{T}$.*

**Proof** **of Theorem 1.** An optimal sensor fault and delay tolerant control law will be given, in which the sensor fault state φ(k) will be a part of the feedforward component.

Applying the optimal control theory, the optimal sensor fault and delay tolerant control law can be as

(21) $$u\left(k\right)=-R_{1}^{-1}\left[B_{1}^{T}\lambda \left(k+1\right)-Q_{zu}^{T}z\left(k\right)-Q_{\varphi u}^{T}\varphi \left(k\right)\right],$$

where λ(k) satisfies the following TPBV problem

(22) $$\left\{\begin{array}{c} z\left(k+1\right)=\bar{A}z\left(k\right)-B_{1}R_{1}^{-1}B_{1}^{T}\lambda \left(k+1\right)+B_{1}R_{1}^{-1}Q_{\varphi u}^{T}\varphi \left(k\right),\;z\left(0\right)=z_{0},\\ \lambda \left(k\right)=Q_{1}z\left(k\right)+Q_{2}\varphi \left(k\right)+{\bar{A}}^{T}\lambda \left(k+1\right),\;\lambda \left(\infty \right)=0. \end{array}\right.$$

λ(k) can be written in the following form

(23) $$\lambda \left(k\right)=P_{1}z\left(k\right)+P_{2}\varphi \left(k\right).$$

By referring to (21) and the second Formula of (22), the optimal controller can be obtained, which is given by

(24) $$u^{*}\left(k\right)=-R_{1}^{-1}\left(B_{1}^{T}{\bar{A}}^{-T}\left(P_{1}-Q_{1}\right)-Q_{zu}^{T}\right)z\left(k\right)+\left(B_{1}^{T}{\bar{A}}^{-T}\left(P_{2}-Q_{2}\right)-Q_{\varphi u}^{T}\right)\varphi \left(k\right).$$

Substitution of (23) into the second Formula of (22) results in

(25) $$P_{1}z\left(k\right)+P_{2}\varphi \left(k\right)=Q_{1}z\left(k\right)+Q_{2}\varphi \left(k\right)+{\bar{A}}^{T}P_{1}z\left(k+1\right)+{\bar{A}}^{T}P_{2}G_{s}\varphi \left(k\right),$$

and substitution of (21) into the first formula of (22) results in

(26) $$z\left(k+1\right)=\bar{A}z\left(k\right)-B_{1}R_{1}^{-1}B_{1}^{T}\left(P_{1}z\left(k+1\right)+P_{2}G_{s}\varphi \left(k\right)\right)+B_{1}R_{1}^{-1}Q_{\varphi u}^{T}\varphi \left(k\right).$$

Notice that (25) and (26) do not involve λ(k) and thus we have eliminated λ(k).

Rearranging (26), we have

(27) $$z\left(k+1\right)={\left(I+B_{1}R_{1}^{-1}B_{1}^{T}P_{1}\right)}^{-1}\left[\bar{A}z\left(k\right)+\left(B_{1}R_{1}^{-1}Q_{\varphi u}^{T}-B_{1}R_{1}^{-1}B_{1}^{T}P_{2}G_{s}\right)\varphi \left(k\right)\right].$$

By substituting (27) into (25), we obtain

(28) $$\begin{array}{c} P_{1}z\left(k\right)+P_{2}\varphi \left(k\right)\\ =Q_{1}z\left(k\right)+Q_{2}\varphi \left(k\right)+{\bar{A}}^{T}P_{1}z\left(k+1\right)+{\bar{A}}^{T}P_{2}G_{s}\varphi \left(k\right)\\ =\left(Q_{1}+{\bar{A}}^{T}P_{1}{\left(I+B_{1}R_{1}^{-1}B_{1}^{T}P_{1}\right)}^{-1}\bar{A}\right)z\left(k\right)+\\ \;\left(Q_{2}+{\bar{A}}^{T}P_{2}G_{s}+{\bar{A}}^{T}P_{1}{\left(I+B_{1}R_{1}^{-1}B_{1}^{T}P_{1}\right)}^{-1}\left(B_{1}R_{1}^{-1}Q_{\varphi u}^{T}-B_{1}R_{1}^{-1}B_{1}^{T}P_{2}G_{s}\right)\right)\varphi \left(k\right). \end{array}$$

By using the matrix inversion lemma

(29) $${\left(I+B_{1}R_{1}^{-1}B_{1}^{T}P_{1}\right)}^{-1}=I-B_{1}{\left(R_{1}+B_{1}^{T}P_{1}B_{1}\right)}^{-1}B_{1}P_{1},$$

we obtain the Riccati matrix Equation (19) and Stein matrix Equation (20).

The existence and the uniqueness of the optimal sensor fault and delay tolerant control scheme (24) are equivalent to the ones of the Riccati matrix Equation (19) and Stein matrix Equation (20). In fact, Assumptions 1 and 3 guarantee that the Riccati matrix Equation (19) has a unique positive semi-definite solution $P_{1}$. Under Assumption 2, we have

(30) $$∥\lambda_{i}\left({\bar{A}}^{T}\left(I-P_{1}{\left(I+B_{1}R_{1}^{-1}B_{1}^{T}P_{1}\right)}^{-1}B_{1}R_{1}^{-1}B_{1}^{T}\right)\right)∥\times ∥\lambda_{j}\left(G_{s}\right)∥<1.$$

Thus, from Lemma 1, the Stein matrix Equation (20) has a unique solution $P_{2}$. Therefore, the proposed feedforward and feedback optimal sensor fault and delay tolerant control Scheme (24) is existence and uniqueness.

However, the optimal control Scheme (24) is a physically unrealizable vector caused by the feedforward item φ(k). In order to make the proposed sensor fault and delay tolerant control scheme more practical and diagnose the sensor fault more accurately, the sensor fault diagnoser (17) is proposed on a designed observer.

In order to obtain the fault diagnoser error equation, let us subtract the first formula of (17) from (3)

(31) $$\varphi \left(k+1\right)-\tilde{\varphi }\left(k+1\right)=\left(G_{s}-K_{e}F_{s}\right)\left(\varphi \left(k\right)-\tilde{\varphi }\left(k\right)\right).$$

Now defining the difference between φ(k) and $\tilde{\varphi }\left(k\right)$ as the error e(k)

(32) $$e\left(k\right)=\varphi \left(k\right)-\tilde{\varphi }\left(k\right),$$

(32) can be rewritten as

(33) $$e\left(k+1\right)=\left(G_{s}-K_{e}F_{s}\right)e\left(k\right).$$

Then, by using the designed fault diagnoser (31) with reasonable feedback gain matrix $K_{e}$, the proposed sensor fault and delay tolerant control Scheme (18) is obtained. ☐

**Remark** **1.** For the fault diagnoser error Equation (33), the dynamic behavior of the error signal is determined by the eigenvalues of $G_{s}-K_{e}F_{s}$. The value of $K_{e}$ will be designed to make the fault diagnoser error e(k) tend to zero with adequate speed regardless of the values of $\varphi (h_{s})$ and $\tilde{\varphi }\left(h_{s}\right)$. In order to obtain a quick response to sensor fault, all eigenvalues of $G_{s}-K_{e}F_{s}$ are chosen as to be zero to obtain a deadbeat response.

## 4. Simulation Result

In this section, the proposed fault diagnoser will be applied to two sensor faults with attenuation and oscillation characters first. In what follows, the effectiveness of the proposed feedback and feedforward sensor fault and delay tolerant control scheme will be investigated under different sensor delays.

### 4.1. Analysis of Designed Fault Diagnosis for Different Types of Sensor Faults

In order to show the validity of the designed fault diagnoser, two sensor faults with attenuation and oscillation characters are analyzed in this subsection. For one case in which the sensor fault is in the form of an attenuation type, the matrices of $G_{s}$ and $F_{s}$ are defined as

(34) $$G_{s}=\left[\begin{array}{cc} 0.9810 & 0.1001\\ -0.1001 & 0.9305 \end{array}\right],F_{s}=\left[\begin{array}{cc} 1 & 0 \end{array}\right].$$

In addition, for another case in which the sensor fault is in the form of an oscillation type, the matrices $G_{s}$ and $F_{s}$ are defined as

(35) $$G_{s}=\left[\begin{array}{cc} 0.9850 & 0.0995\\ -0.2985 & 0.9850 \end{array}\right],F_{s}=\left[\begin{array}{cc} 1 & 0 \end{array}\right].$$

Noted Remark 1, the desired eigenvalues of the fault diagnoser (16) set as

(36) z=0.1+j0.1,z=0.1−j0.1.

Based on the Ackermann formula, the fault diagnoser gain matrices are designed as

(37) $$K_{a}=\left[\begin{array}{cc} 1.7115 & 6.8902 \end{array}\right],\;K_{o}=\left[\begin{array}{cc} 1.7700 & 7.6736 \end{array}\right],$$

where fault diagnoser feedback gain matrixes $K_{a}$ and $K_{o}$ are defined for the attenuation sensor fault (34) and the oscillation sensor fault (35), respectively.

**Figure 1:**
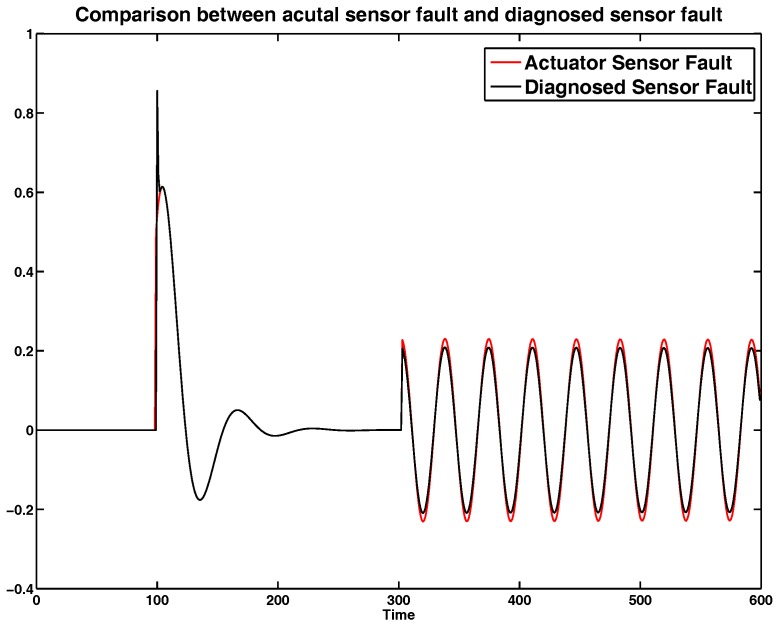
Diagnosed actuator fault ${\tilde{f}}_{s}\left(k\right)$ from the fault diagnoser.

**Figure 2:**
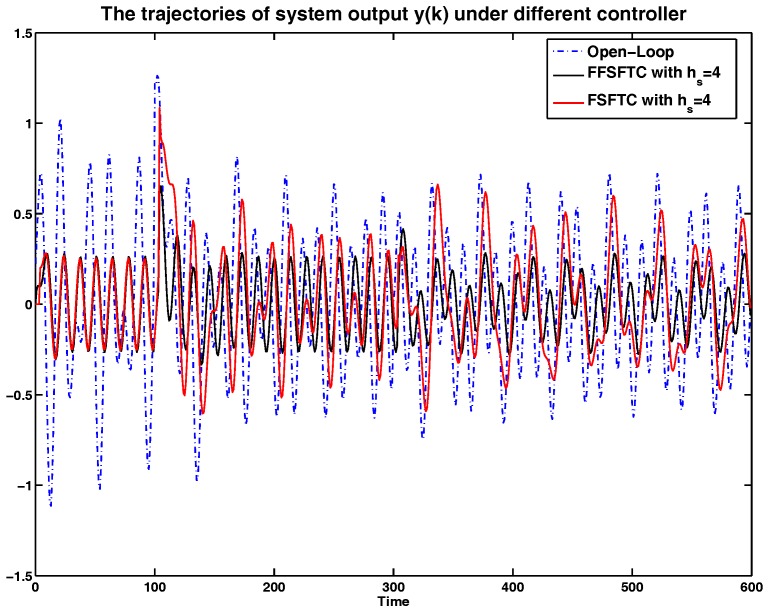
The comparison of system output *y* under open-loop systems, the proposed feedforward and feedback sensor fault tolerant controller (FFSFTC), and classic feedback sensor fault tolerant controller (FSFTC) with $h_{s}=4$.

Setting the occurrence times of sensor faults (34) and (35) as k=100 and k=300, the curves of comparison result between actual sensor fault and diagnosed sensor fault are shown in Figure 1, in which the diagnosed sensor fault is from the designed fault diagnoser (18). Meanwhile, the curve of observed error between actual and diagnosed sensor fault is shown in Figure 2. One can see that the values of observed error approach zero. While the sensor faults happen, the observed error appears to show very small fluctuations. After that, it will converge to small values rapidly. Then, the designed sensor fault can approximate the actual sensor fault rapidly.

Therefore, no matter what the type of sensor fault, the diagnosed fault ${\tilde{f}}_{s}\left(k\right)$ can be converged to the actual sensor fault, which is the necessary prerequisite to apply the sensor fault and delay tolerant control Scheme (18).

### 4.2. Analysis of Designed FFSFTC under Different Controllers

In this subsection, the validity and reliability of the proposed FFSFTC (18) will be proved under different sensor delays.

Consider system (1) with the following matrices:

(38) $$\begin{array}{c} A=\left[\begin{array}{cc} 1 & 0.45\\ -0.2 & 0.9 \end{array}\right],B=\left[\begin{array}{c} 0.3\\ 0.5 \end{array}\right],D_{v}=\left[\begin{array}{c} 0.2084\\ 0.1541 \end{array}\right],\\ C=\left[\begin{array}{cc} 1 & 0 \end{array}\right],D_{s}=2,x_{0}={\left[\begin{array}{cc} 3 & 2 \end{array}\right]}^{T}, \end{array}$$

where the initial value is $x_{0}={\left[\begin{array}{cc} 0.21 & 0.12 \end{array}\right]}^{T}$. The external disturbance (2) is described as the following sinusoidal form:

(39) $$G_{v}=\left[\begin{array}{cc} 0.8944 & 0.4\\ -0.5 & 0.8944 \end{array}\right],H_{v}=\left[\begin{array}{cc} 1 & 0 \end{array}\right],w_{0}=\left[\begin{array}{cc} 0.5 & 0 \end{array}\right],$$

with $w_{0}={\left[\begin{array}{cc} 0.2 & 0.1 \end{array}\right]}^{T}$.

In order to validate the effectiveness of the proposed control scheme, the following classic feedback sensor fault tolerant controller (FSFTC) is used to compare with the proposed FFSFTC (18), which is given by

(40) $$u\left(k\right)=-R_{1}^{-1}\left(B_{1}^{T}{\bar{A}}^{-T}\left(P_{1}-Q_{1}\right)-Q_{zu}^{T}\right)z\left(k\right),$$

where the feedforward item in (18) is eliminated.

Meanwhile, the sensor delays are set as $h_{s}=2,4,6$ under this simulation to show the reliability of the proposed control scheme. The performance index (4) is with Q=1 and R=1.

**Figure 3:**
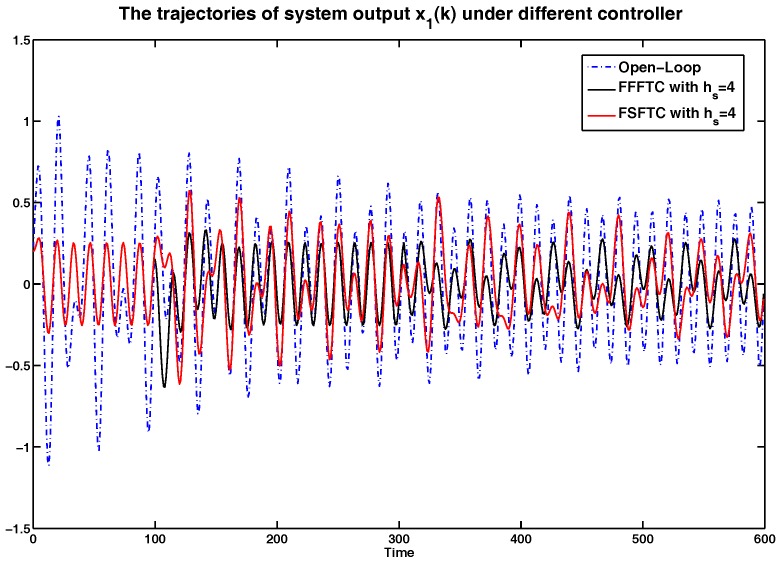
The comparison of system state $x_{1}$ under open-loop system, FFSFTC and FSFTC with $h_{s}=4$.

**Figure 4:**
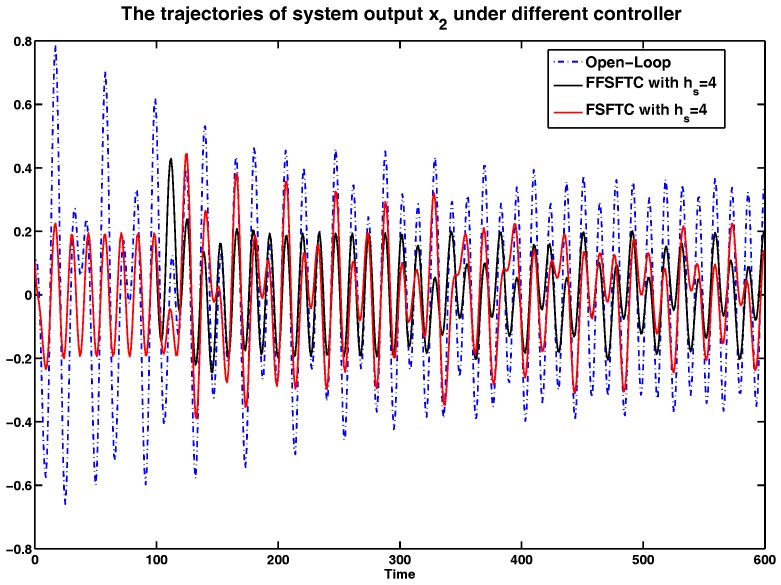
The comparison of system state $x_{2}$ under open-loop system, FFSFTC and FSFTC with $h_{s}=4$.

In order to show the simulation results clearly, the curves of system output *y* and system states $x_{1}$ and $x_{2}$ are represented in Figure 2, Figure 3 and Figure 4 compared with open-loop systems and FSFTCs. It is obvious that system output *y*, system states $x_{1}$ and $x_{2}$ can be converged to smaller values under FSFTC and the proposed FFSFTC. By observing the tendency of change at the occurrence times k=100 and k=300, the proposed FFSFTC can respond to the sensor faults better than FSFTCs. This is caused by the elimination of feedforward item ${\tilde{f}}_{s}\left(k\right)$ in FSFTC. Meanwhile, system output *y* and system states $x_{1}$ and $x_{2}$ can not converge to zero caused by the sensor fault $f_{s}\left(k\right)$ and external disturbance v(k). However, at a later stage, the oscillation of system output derives from the oscillation sensor fault $f_{s}\left(k\right)$ and persistent disturbance v(k). Regardless of the values of sensor delays $h_{s}$, it can be seen that the system output can significantly reduce and converge to small values.

**Figure 5:**
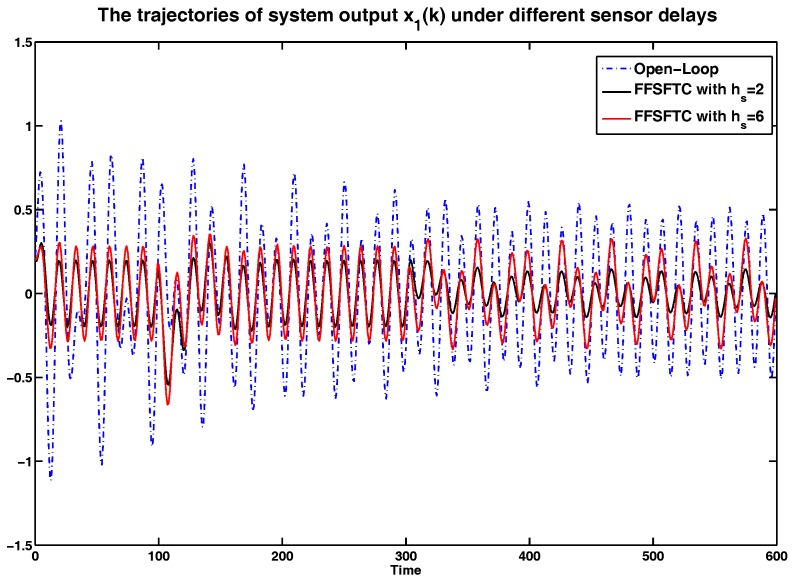
The comparison of system state $x_{1}$ under sensor delays with $h_{s}=2,6$.

**Figure 6:**
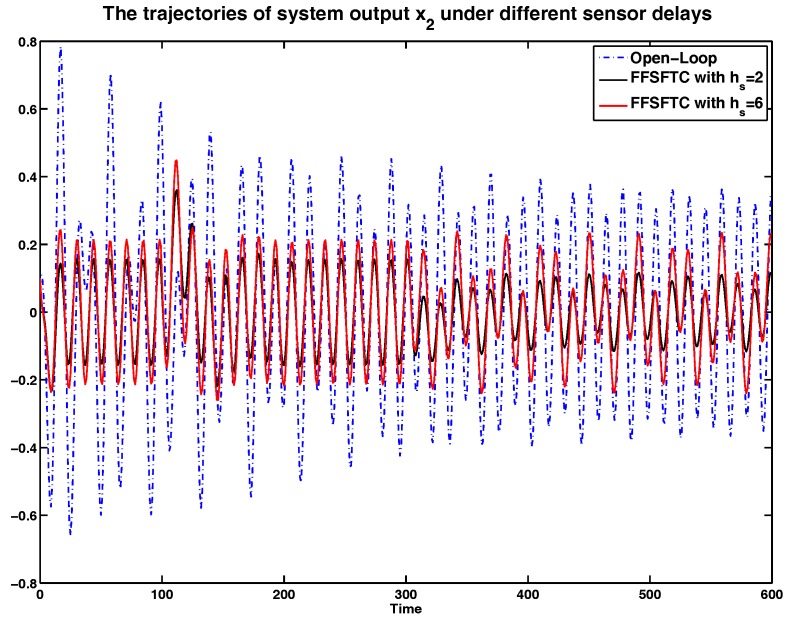
The comparison of system state $x_{2}$ under sensor delays with $h_{s}=2,6$.

**Figure 7:**
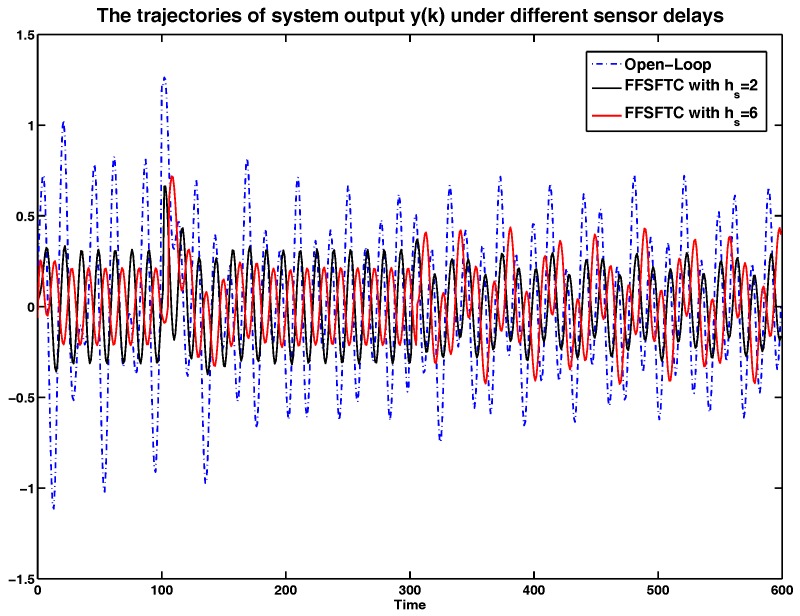
The comparison of system output *y* under sensor delays with $h_{s}=2,6$.

In order to show the sensor delay tolerant ability of proposed FFSFTC, the simulation results will be analyzed under different sensor delays. Considering the system with (38), the sensor faults with (34) and (35), and the external disturbance (39), the curves of system output *y*, and system states $x_{1}$ and $x_{2}$ are represented in Figure 5, Figure 6 and Figure 7 under the open-loop system and sensor delays $h_{s}=2,6$. It is obvious that the values of *y*, $x_{1}$ and $x_{2}$ can be converged to small values compared to the open-loop system. Meanwhile, the proposed FFSFTC can offset the sensor faults effectively.

Furthermore, the system is under persistent disturbance. Therefore, the proposed FFSFTC could offset the vibration caused by the persistent disturbance v(k). It means that, as time passes, the system states could converge to small values. From Figure 2, Figure 3, Figure 4, Figure 5, Figure 6 and Figure 7, one can see that the system states $x_{1}$ and $x_{2}$ cannot converge to zero caused by the persistent disturbances. Fortunately, the system states $x_{1}$ and $x_{2}$ can converge to small values under sensor faults, sensor delays and external disturbance. This illustrates that the proposed control scheme can offset the persistent disturbances effectively.

To more intuitive to valid the abilities of sensor delay tolerance and offsetting the external disturbance, the comparisons of RMS (Root Mean Square) values for system output *y*, system state $x_{1}$ and $x_{2}$ are shown in Table 1, Table 2 and Table 3. Meanwhile, the comparison results of performance indices among the open-loop system and the closed-loop system are shown in Table 4. For example, under sensor faults $h_{s}=2,4,6$, the values of performance indices are decreased by 64.06%,65.14% and 61.71% under FFSFTC compared to the open-loop systems, respectively. Correspondingly, the values of performance indices are decreased by 32.63%,41.84% and 49.01% under FSFTC compared to the open-loop systems. One can see that the above indices can be reduced obviously under proposed FFSFTC compared to those of the open-loop systems and FSFTCs. At the same time, no matter how much the sensor faults, the performance indices could be converged to ones around 0.06. Therefore, the validity of the proposed sensor fault tolerant control Scheme (18) is proved.

In summary, it can be observed that the proposed sensor fault and delay tolerant control scheme can significantly tolerate the influence from sensor fault and delay, offset the persistent disturbances, and improve the control performance.

## 5. Conclusions

In order to eliminate the influence from the sensor fault and external disturbance, a fault diagnoser has been designed and a sensor fault and delay tolerant control scheme has been proposed in this paper. By introducing a vector transformation, an available delay-free system output was designed and an augmented system was constructed by combining the control system and external disturbances. Then, a sensor fault diagnoser was designed to diagnose the sensor fault. A physically realizable sensor delay and fault tolerant controller was proposed, in which the gain matrices were derived from a Riccati equation and a Stein equation, respectively. Taking the different types of sensor faults into consideration, simulation results have shown that the proposed controller can have the abilities of offsetting external disturbances, tolerating the sensor fault and delay, and improving the control performance under different sensor delays, respectively.

In this paper, the results presented are based on the fact that the external disturbance and sensor fault have well known dynamic characteristics. Meanwhile, it is assumed that the sensor fault has a constant value. In fact, the dynamic characteristics of the external disturbance and sensor fault are unknown, even partially. In addition, the sensor delay usually is with random value. Actually, many control systems usually are with actuator fault and delays. Therefore, the future work may include the following aspects:

(1) A more general sensor and actuator fault tolerant control problem should be investigated to offset the external disturbance, sensor fault, and actuator fault with unknown characteristics.
(2) Taking the uncertain actuator and sensor delays into consideration, the controller could be designed to improve the control performance.

## Figures and Tables

**Table 1:** The comparison root mean square (RMS) values of system output *y* under different controllers.

Sensor Delay	Open-Loop	FFSFTC	FSFTC
$h_{s}=2$	0.4076	0.1884 (−53.79%)	0.2083 (−48.89%)
$h_{s}=4$	0.4076	0.1792 (−56.03%)	0.2616 (−35.82%)
$h_{s}=6$	0.4076	0.2095 (−48.60%)	0.2962 (−27.33%)

**Table 2:** The comparison RMS values of system state $x_{1}$ under different controllers.

Sensor Delay	Open-Loop	FFSFTC	FSFTC
$h_{s}=2$	0.3834	0.1323 (−65.49%)	0.2083 (−45.67%)
$h_{s}=4$	0.3834	0.1704 (−55.55%)	0.2079 (−45.77%)
$h_{s}=6$	0.3834	0.1954 (−49.03%)	0.2416 (−36.98%)

**Table 3:** The comparison RMS values of system state $x_{2}$ under different controllers.

Sensor Delay	Open-Loop	FFSFTC	FSFTC
$h_{s}=2$	0.2713	0.1019 (−62.44%)	0.1203 (−55.66%)
$h_{s}=4$	0.2713	0.1302 (−52.01%)	0.1501 (−44.67%)
$h_{s}=6$	0.2713	0.1447 (−46.66%)	0.1722 (−36.53%)

**Table 4:** The comparison results of performance indices under different sensor delays.

Sensor Delay	Open-Loop	FFSFTC	FSFTC
$h_{s}=2$	0.1661	0.0597 (−64.06%)	0.1119 (−32.63%)
$h_{s}=4$	0.1661	0.0579 (−65.14%)	0.0966 (−41.84%)
$h_{s}=6$	0.1661	0.0636 (−61.71%)	0.0847 (−49.01%)
